# Commercially Available Textiles as a Scaffolding Platform for Large-Scale Cell Culture

**DOI:** 10.1155/2023/2227509

**Published:** 2023-03-01

**Authors:** Tarun Agarwal, Sheri-Ann Tan, Shanmuga Sharan Rathnam Vuppaladadium, Tanmayi Sajja, Tapas Kumar Maiti

**Affiliations:** ^1^Department of Biotechnology, Indian Institute of Technology, Kharagpur, West Bengal-721302, India; ^2^Department of Bio-Technology, Koneru Lakshmaiah Education Foundation, Vaddeswaram, AP, India; ^3^Department of Bioscience, Faculty of Applied Sciences, Tunku Abdul Rahman University of Management and Technology, Kuala Lumpur 53300, Malaysia; ^4^Department of Biotechnology and Medical Engineering, National Institute of Technology, Rourkela, Odisha, India

## Abstract

The present study outlines the evaluation of textile materials that are currently in the market for cell culture applications. By using normal LaserJet printing techniques, we created the substrates, which were then characterized physicochemically and biologically. In particular, (i) we found that the weave pattern and (ii) the chemical nature of the textiles significantly influenced the behaviour of the cells. Textiles with closely knitted fibers and cell adhesion motifs, exhibited better cell adhesion and proliferation over a period of 7 days. All the substrates supported good viability of cells (>80%). We believe that these aspects make commercially available textiles as a potential candidate for large-scale culture of adherent cells.

## 1. Introduction

Current market demands exploration of new avenues as a cell culture platform that possesses a large scalability [1]. Such platforms could revolutionize the aspects of the adherent cell both in the industrial and medical sectors. Large-scale cultures are primarily needed for vaccine production [2], protein/biopharmaceutics production [3], and they even form a part of various extracorporeal devices such as artificial kidney and livers [4–6]. The technologies available in the market for large-scale cultures include T-flask, packed bed bioreactor, multiplate bioreactor, roller bottles, and microcarriers [7–11]. However, no technology could still address all the challenges (maintenance of an adequate culture environment, scalability, efficiency, and affordability) associated with the large-scale culturing. For example, T-flask and cell factories, multiplate bioreactor, and roller bottles offer a 2D culture environment, which influences the physiology of adherent cells, and the subsequent production of cell-based therapeutics [12, 13]. On the other hand, packed bed bioreactor and microcarrier technologies provide a quasi-3D culture environment, but they are associated with large mass transfer gradients, which compromise their performance [14, 15]. The major bottlenecks of large-scale culture of adherent cells are—(i) to provide large surface area keeping the footprint of the bioreactor within reasonable dimensions, (ii) overcoming the mass transfer constraints without compromising culture density, and (iii) the 3D complex microenvironment needed for effective cell functioning, particularly in the case of anchorage-dependent cells.

In this regard, the use of textiles could prove advantageous. Textile refers to a flexible material consisting of a network of fibers, thin threads, or filaments and has major utility in the fashion industries, interior decoration-associated firms, and packaging industries [16]. These threads/fibers form the building block of the fabric and consists of either natural polymers (such as cotton, flax, and silk) or human-made polymers (nylon, poly (*ε*-caprolactone), and polyester) or combination of both. Moreover, threads could be spun into the fabric using various methods like weaving, knitting, braiding, and others, depending upon their utility [16]. Recently, they are also being employed for tissue engineering and developing large-scale cell culture platforms due to advantages in terms of high surface to volume ratio, 2.5/3D microarchitecture, and well-established fabrication process [17–19]. Until date, the woven textiles have been used for culturing various cell types including hepatocytes [20, 21], cardiomyocytes [22, 23], tenocytes [16, 24], endothelial cells [16] (refer supplementary Table S1). However, all the studies reported to date, have utilized the in-lab engineered textile fabric, which involve high investment and the use of sophisticated instrumentation.

Here, we raise a question that can commercially available textile materials (generally used for preparing wearable clothes) be used for large-scale culture of adherent cells? With this perspective, we evaluated various textile materials for cell culture applications. We are particularly interested in these developed fabrics due to their affordability,bringing down the overall cost of establishing large-scale cultures by a significant amount.

## 2. Evaluating Textiles for Cell Culture Applications: Materials and Methods

### 2.1. Materials

Textile samples were collected from a cloth house in Lucknow, Uttar Pradesh, India. Calcein-AM and ethidium homodimer were procured from Invitrogen, India. Minimum essential medium (MEM), fetal bovine serum (FBS), antibiotic-antimycotic solution, and Trypsin-EDTA were bought from HiMedia, India. HepG2 cell line was obtained from the National Center for Cell Science (NCCS), Pune, India.

### 2.2. Methods

#### 2.2.1. Preparation of Textile-Based Devices

**Figure 1:**
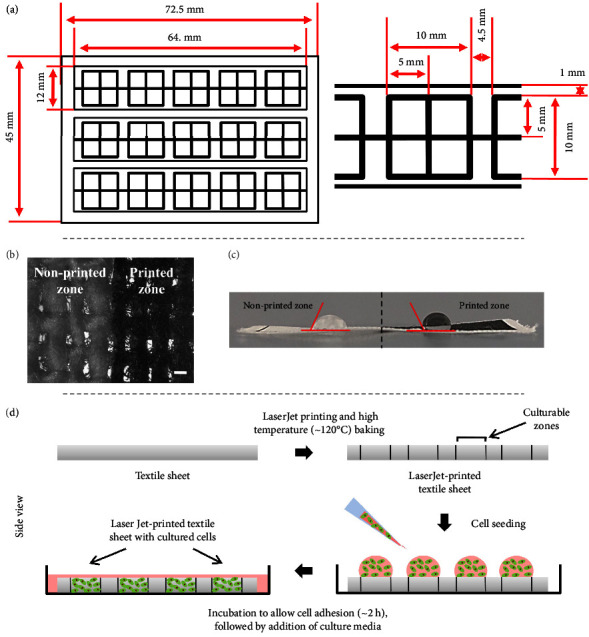
Textile-based devices for cell culture applications. (a) Design specifications of the devices. (b) Micrographs of the textile-based devices showing the differences in the printed and nonprinted zones. The printed zones showed the presence of coating of melted toner ink particles (40x magnification and scale bar: 200 *μ*m). (c) Contact angle measurement of the printed (hydrophobic, contact angle: ∼112.5°) and nonprinted (hydrophilic, contact angle: ∼70.5°) zones of the textile devices. (d) Schematics demonstrating the preparation and cell seeding on the textile devices. Representative images for cell seeding on the textile device have been provided in supplementary figure S3.

For the preparation of the textile-based devices, the design was created using Microsoft PowerPoint software as described by Agarwal et al. (Figure 1(a)) [25]. Thereafter, the design was printed on the textile using a LaserJet printer (HP). Postprinting, the devices were baked at ∼120 °C for 3-4 min to ensure formation of a uniform hydrophobic barrier. The devices were then cut and autoclaved.

#### 2.2.2. Morphological Characterization of Textile-Based Devices

The surface morphology of the substrates was observed by scanning electron microscopy (SEM, ZEISS) after sputter-coating the samples. The visualization of the samples was done at 20 kV of voltage. The contact angle analysis was done to evaluate the hydrophobicity of the textile samples. For this, we prepared the devices as discussed above. Then, a 30 *μ*L drop of water was placed on the surface. After 2-3 min, images were taken using a camera (Motorola G60), and contact angle was analyzed using angle tool of WCIF ImageJ software.

#### 2.2.3. Cell Culture on the Textile-Based Devices

We selected HepG2 as a model cell line for evaluating the biological compatibility of the textile material. The cells were maintained in MEM supplemented with 10% FBS and 1× antibiotic-antimycotic solution at 37°C in a humidified CO_2_ (5%) incubator. Regular passaging of cell was done once they reach a confluency of ∼80%.

For cell seeding, the textile-based devices were placed in individual well of a 24-well plate and air-dried. Thereafter, 10 *μ*l of cell suspension containing 7.5 × 10^3^ cells was added onto each culturable area of the devices, followed by 2.5 h incubation at 37°C in a humidified CO_2_ incubator. Postseeding, precaution was taken to prevent drying of the devices. Thereafter, 600 *μ*L of complete culture media was added to each well. The set-up was then placed at 37°C in a humidified CO_2_ incubator with a regular media change every 2-3 days.

The viability of cells on different textile-based devices was assessed using calcein-AM/ethidium homodimer-based live/dead staining, following manufacturer instructions, and further visualized using fluorescence microscopy (Olympus IX71).

## 3. Results and Discussion

### 3.1. Preparation of Textile-Based Substrates

A wide range of reports exists that have used textiles as cell culture platforms. These studies have prepared textiles (with specific weaving pattern) using in-house developed polymeric threads. However, no study exists, to date, that evaluates commercially available textiles for cell culture applications. A major advantage of using these substrates is their cost-effectiveness, easy availability, and availability in wide range of formats and polymeric composition.

For the first time, we evaluated the suitability of the commercially available textiles, such as cotton (including voile (VC) and rubia cotton (RC)), linen (L), silk (S), and nylon (N) for cell culture applications. We demonstrate that the textiles could easily be printed using simple LaserJet-based strategy to create barriers to restrict the cells in the confined area. It could further be utilized to achieve spatially localized cell distribution. In this strategy, we first created the design using Microsoft PowerPoint software (version 2019) and then printed on the textile with a traditional LaserJet printer (HP LaserJet 1020 Plus Printer). Postprinting, substrates were baked to form proper hydrophobic barrier via melting of toner ink particles. This resulted in the formation of 2 discrete zones on the textile-hydrophobic printed zones (black in color) and white/pale yellow nonprinted hydrophilic zones. The scanning electron micrographs also showed the presence of a coating of melted toner ink particles, while nonprinted zone remained unaltered (Figure 1(b)). The contact angle analysis also validated the hydrophobicity of the printed zones (contact angle: ∼112.5°) as compared to nonprinted ones (contact angle: ∼70.5°) (Figure 1(c)). Owing to their hydrophobicity, the printed zones would restrict the cell adhesion as well as migration. On the other hand, at the nonprinted zones, cell can easily interact and adhere onto the substrates.

Further, the devices were cut, sterilized, and used for cell seeding (Figure 1(d)). Here, it is also important to mention that neither the fabrication nor the sterilization processes caused any observable damage to the textile substrates.

### 3.2. Morphological Characterization of the Textile Substrates

**Figure 2:**
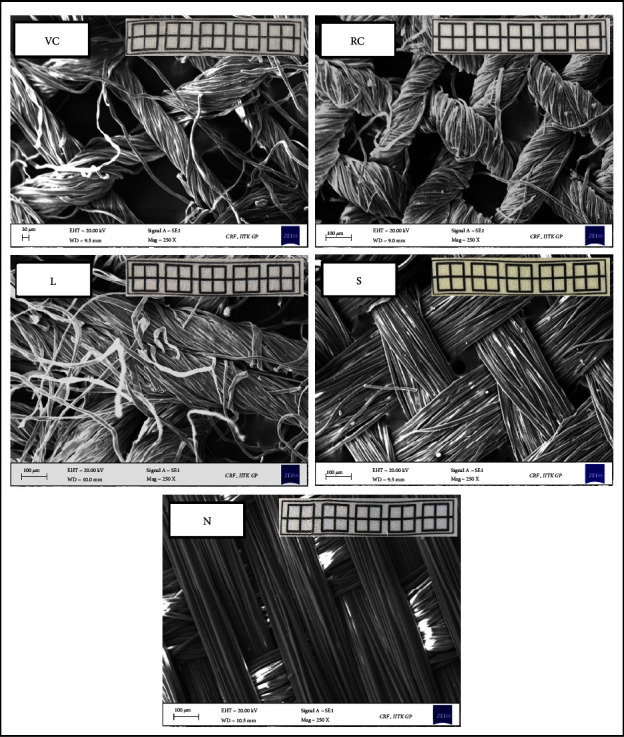
Scanning electron micrographs of the selected textiles (scale bar: 100 *μ*m). Inset: camera images of the prepared textile-based substrates.

The textile substrates were characterized for their surface topology. The results revealed that all the samples formed a porous matrix, with VC having the highest pore size (Figure 2). Notably, all textile substrates had a plain weaving pattern except nylon (satin weaving pattern). We also observed that the thread used for weaving VC, RC, and L samples consisted of a bundle of cellulosic fibrils, while no such bundling was present in S and N substrates.

### 3.3. Cell Culture on the Textile Substrates

**Figure 3:**
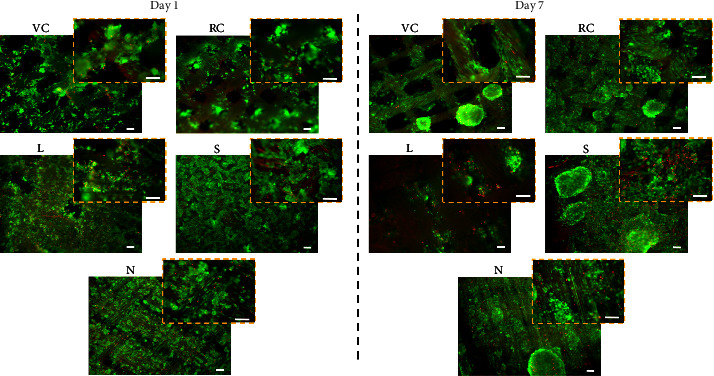
HepG2 cell culture on the textiles. Live/dead images of cells cultured on selected textiles at day 1 and 7 at magnifications, 40x (scale bar: 200 *μ*m) and 100x (inset image; scale bar: 100 *μ*m).

For evaluating the cell culture potential of the textile substrates, we seeded HepG2 cells, as demonstrated in Supplementary Figure S3. The results demonstrated that cells adhered on all the substrates; however, their adhesion was critically dependent on—(i) weaving pattern and (ii) the chemical nature of the textiles. Precisely, close knitting of the textile threads were associated with higher cell adhesion, as observed in the case of samples N, S, and L. On the other hand, sample S, containing cell-adhesive motifs supported better cell adhesion and spreading compared to other matrices (Figure 3). Interestingly, despite the fact that VC, RC, and L are mainly composed of cellulose, substrate L supported higher cellular adhesion, suggesting that cell adhesion was modulated by close packing of the textile threads. After day 1 of cell seeding, viable cell populations (>90%) were observed on all the substrates. The major cell population adhered as cell clusters, with some cells spreading along the fibrils of the textiles. Higher numbers of spread cells were evident in nylon and silk, followed by other cotton-based textiles.

After day 7 of culture, we observed the presence of a viable cell population on all the textiles, with a significant increase in cell number on all the substrates. Moreover, cells achieved a spread conformation, mostly aligned along the textile fibrils (Figure 3). Notably, RC, S, and N samples supported the highest cellular proliferation; whereas samples L and S showed relatively higher cell death. On substrate S, cell death might be due to overcrowding, while on substrate L, material composition and chemical treatments (usually employed during the textile preparation) might have impacted cell viability.

Together, these findings point to the suitability of commercially available textiles for adherent cell culture, and if put into practice, they could significantly lower the cost of large-scale cell culture.

## 4. Conclusion

**Figure 4:**
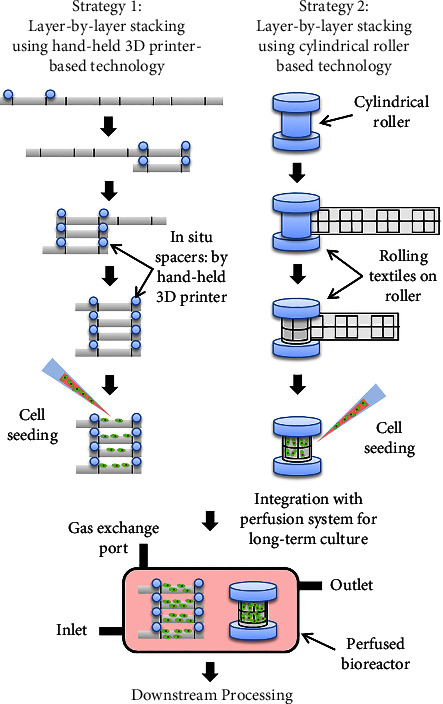
Strategies for large-scale cell culture using textile substrates.

In the present study, we showed that the commercially available textiles (primarily aimed for preparing wearable clothes) could act as a suitable cell scaffolding platform. The fibrillar architecture of textiles allows adequate cellular adhesion, spreading, and proliferation. Moreover, >80% of cells maintained their viability over the culture period. The fabricated devices could further be extended for the development of large-scale culture platforms using simple layer-by-layer stacking strategies, as demonstrated in Figure 4. Besides, various bio-functionalization approaches could be integrated with the textiles to improve their biological properties, thereby modulating the functional properties of the cultured cells. Recent advancements made in the paper-based technologies for cell culture applications could provide a ray of hope for further progress in this field [25–28].

As far as economic aspects are concerned, investments made on the suitable substrates for large-scale culturing cell are one of the crucial contributors. A major focus is on choosing a substrate that can provide a higher surface area to volume ratio for better cell and product yield. In this regard, let us consider the example of microcarriers, one of the most opted substrates for large-scale culture. Microcarriers (from Sartorius®) provides a surface area of ∼360 cm^2^/g and cost atleast USD 300–1000. In contrast, according to Ko et al. textiles with a fiber diameter of ∼90–100 *μ*m can also provide a surface area of 300–400 cm^2^/g [29]; however, the relative cost is considerably low (∼USD 2–5), resulting in an almost 100-folds cost reduction for procuring substrates. Thus, if we consider all other parameters same, it is quite possible to lower the overall cost of large-scale culture of adherent cells using commercially available textiles.

Notably, one must also keep in mind the disadvantages associated with textiles. Commercially available textiles undergo various treatments that may significantly influence the properties of the textile itself and also affect cellular responses.

## Data Availability

The data supporting the findings of this study are available from the corresponding author upon reasonable request.

## Supplementary Materials

Supplementary Materials Supplementary file method and representative images for the chemical characterization of textile matrices, bright field image showing distinguishable characteristics of printed and nonprinted zones, and method for cell seeding on textile devices. Table compiling different research studies in this domain is also provided.
