# Supplementary material for: Commercially Available Textiles as a Scaffolding Platform for Large-Scale Cell Culture
Source: Int J Biomater. 2023 Mar 1;2023:2227509. doi: 10.1155/2023/2227509 (PMC9995198; doi:10.1155/2023/2227509)
Supplement: Supplementary Materials — Supplementary file method and representative images for the chemical characterization of textile matrices, bright field image showing distinguishable characteristics of printed and nonprinted zones, and method for cell seeding on textile devices. Table compiling different research studies in this domain is also provided. [file 2227509.f1.docx]

**Supplementary File**

**Commercially available textiles as a scaffolding platform for large-scale cell culture**

Tarun Agarwal^1,2,^*, Sheri-Ann Tan^3^, Shanmuga Sharan Rathnam Vuppaladadium^4^, Sajja Tanmayi^2^, Tapas Kumar Maiti^1,^*

^1^ Department of Biotechnology, Indian Institute of Technology, Kharagpur, West Bengal – 721302, India

^2^ Department of Bio – Technology, Koneru Lakshmaiah Education Foundation, Vaddeswaram, AP, India

^3^ Department of Bioscience, Faculty of Applied Sciences, Tunku Abdul Rahman University College, 53300 Kuala Lumpur, Malaysia

^4^ Department of Biotechnology and Medical Engineering, National Institute of Technology Rourkela, Odisha, India

* Corresponding authors, Email id: [tarunagarwal@kluniversity.in](mailto:tarunagarwal@kluniversity.in) (TA); [maititapask06@gmail.com](mailto:maititapask06@gmail.com) (TKM)

**Supplementary Methods**

**SM1. Chemical characterization of textile-based devices**

The chemical features of the fabric material were evaluated using X-ray PS (XPS, PHI 5000 Versa Probe III, Physical Electronics) facility under the default settings present at Indian Institute of Technology, Roorkee, Uttarakhand, India under the default settings. Further, we carried out deconvolution analysis (using Origin Pro8.0 software) of the energy peak of C1s at 280-290 eV binding energy to understand the chemical nature of the substrates.

**Supplementary Results and discussion**

**SR1. Chemical analysis**

Chemical characteristics play a crucial role in modulating the behaviour of the cells, cultured on the substrate [1]. In this regard, we employed XPS for understanding the surface chemical characteristics of the selected textiles. We particularly focussed on the C1s peak at 280-290 eV binding energy (**Figure S1**). In all the samples, we observed a single broad peak of C1s between 280-290 eV binding energy which was deconvoluted to get better understanding of existing bond between carbon and other atoms in the substrates. Notably, the energy peak at ~284.3 eV, corresponding to aliphatic hydrocarbon (C-C), was found to be dominant over the other peaks in all the samples. We observed that the C1s spectra of the VC, RC and L samples were similar to each other. Apart from C-C bond, all three samples showed the presence of energy peak at ~286.3 eV that represents the carbon bonded to oxygen forming glycosidic linkage (C-O) [2]. This could be attributed primarily to the basal cellulosic composition of all three samples. In silk (S), we observed energy peaks at 286.3 and 288.4 eV corresponding to ester (C-O) and carboxyl (C=O) linkages respectively [3]. As silk is protein in nature, a signature amide (C-N) energy peak was also present at 285.3 eV [4]. In the nylon substrate, apart from C-C linkage, energy peaks corresponding to carboxyl (C=O) and amide (C-N) were observed at 288.7 eV and 285.7 eV respectively. The amide bond in nylon is formed by the reaction between the its two monomeric units, i.e., adipic acid and hexamethylene diamine [5].


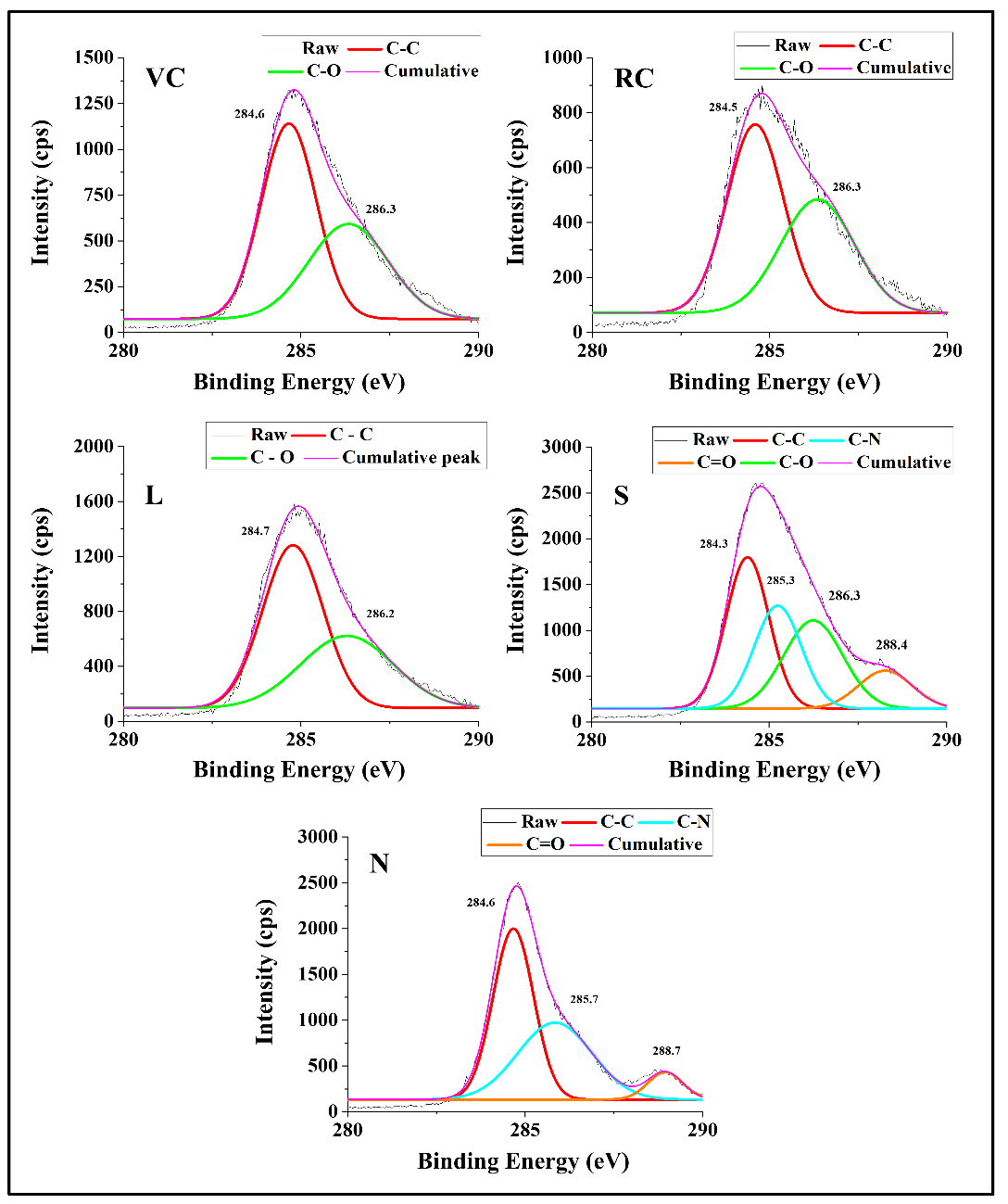


**Figure S1.** High resolution C1s spectra of the textiles. The spectra have been deconvoluted using Origin Pro 8.0 software.


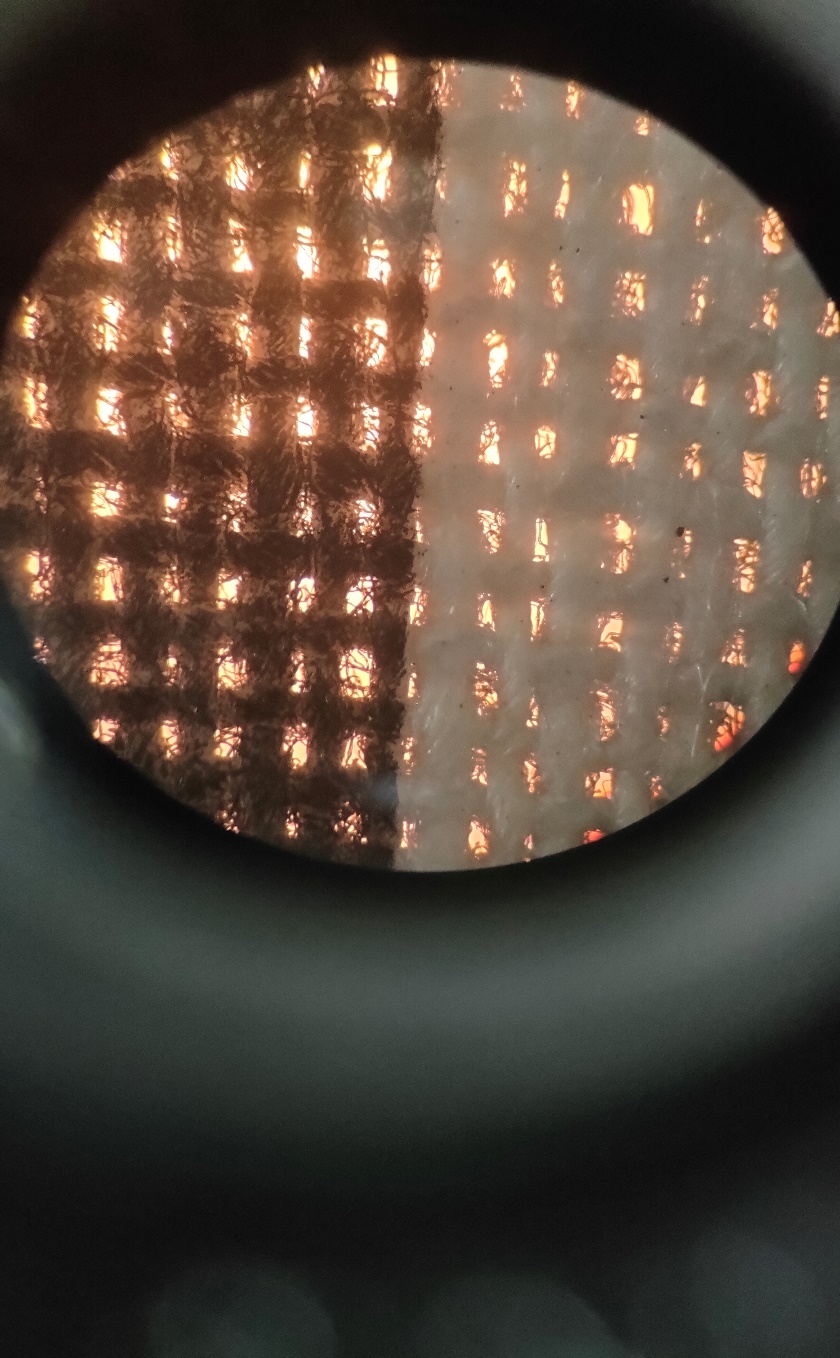


**Figure S2.** Micrographs of textile devices at 40× magnification. Left side – printed zones with black hydrophobic toner ink particle deposition. Right side – non printed zone.


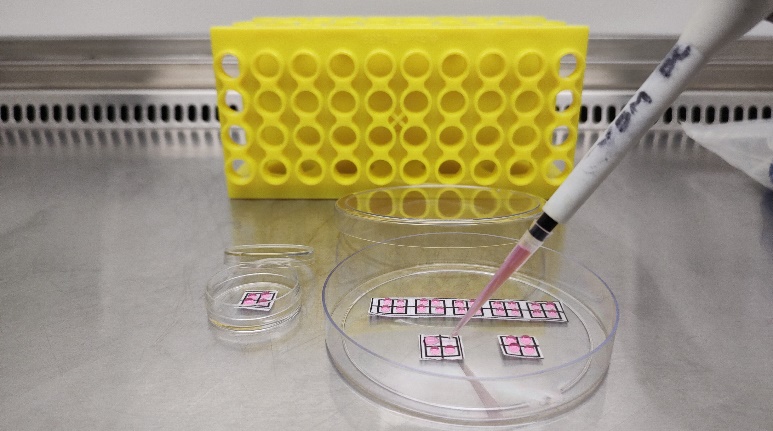

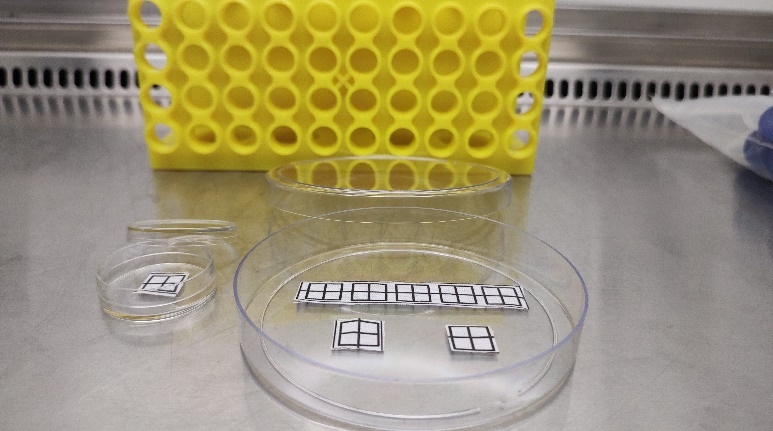


**Figure S3.** Representative images of cell seeding strategy on the textile-based devices.

**Table S1**: Textiles in cell culture-related applications

| **S. No.** | **Textile materials** | **Cells** | **Cellular responses** | **Research paper title** |
| --- | --- | --- | --- | --- |
| 1 | Woven PET fabrics coated with PLGA and PVLA | Mice hepatocytes | Hepatocytes formed smaller aggregates on the fabric scaffolds. | Partially Degradable Film/Fabric Composites: Textile Scaffolds for Liver Cell Culture |
| 2 | Woven PET monofilament fabrics | Primary rat tenocytes | Scaffolds supported adhesion of the cells. Cells (on the scaffolds) upon exposure to mechanical stress showed alignment of F-actin fibres. | Tissue engineered composite of a woven fabric scaffold with tendon cells, response on mechanical simulation in vitro |
| 3 | Woven PGA fabric | Avian tenocytes | Scaffolds were biocompatible and supported adhesion of the tenocytes. The cells were more aligned on woven fabric than non-woven ones. | Use of polyglycolic acid unwoven and woven fibers for tendon engineering in vitro |
| 4 | Woven PCL scaffolds with porcine cartilage-derived ECM | Human adipose-derived stem cells | Scaffolds supported culture and differentiation of stem cells towards chondrogenic lineage. The cells expressed GAG, collagen type II, and chondroitin 4‐sulfate markers of chondrocytes. | Multifunctional Hybrid Three-dimensionally Woven Scaffolds for Cartilage Tissue Engineering |
| 5 | Woven PCL scaffolds | Human bone marrow-derived mesenchymal stem cells | Scaffolds supported a long-term culture of stem cells and supported differentiation towards chondrocytes with expression of sox9 and collagen type II. Mechanical stimulus improved the chondrogenic differentiation and resulted in construct with robust mechanical properties. | In vitro generation of mechanically functional cartilage grafts based on adult human stem cells and 3D-woven poly(ɛ-caprolactone) scaffolds |
| 6 | Woven PCL scaffolds | Human bone marrow-derived mesenchymal stem cells | Scaffolds supported adhesion of stem cells and efficient differentiation towards chondrogenic lineage with the expression of sox9, collagen type II, and aggrecans. | Chondrogenesis and Mineralization During In Vitro Culture of Human Mesenchymal Stem Cells on Three-Dimensional Woven Scaffolds |
| 7 | Woven PCL scaffolds | Human bone marrow-derived mesenchymal stem cells | Scaffolds supported culture and differentiation of stem cells towards chondrogenic lineage. The cells expressed collagen type II and chondroitin 4‐sulfate markers of chondrocytes. | The inhibition by interleukin 1 of MSC chondrogenesis and the development of biomechanical properties in biomimetic 3D woven PCL scaffolds |
| 8 | Woven PCL–fibrin incorporated with fibrin hydrogel | Human adipose-derived stem cells | The constructs supported culture and differentiation of stem cells towards chondrocytes. The differentiated cells expressed collagen type II and chondroitin 4‐sulfate. | Hydrogel-coated textile scaffolds as candidate in liver tissue engineering: II. Evaluation of spheroid formation and viability of hepatocytes |
| 9 | Woven PET scaffolds coated with chitosan and collagen | Primary rat hepatocytes and HepG2 cells | The scaffolds supported attachment and viability of the cells. The cells primarily attained spheroidal morphology and exhibited expression of hepatocyte marker, albumin. The functional aspects of the cells were greatly influenced by the composition of collagen and chitosan coating. | Hydrogel-coated textile scaffolds as candidate in liver tissue engineering: II. Evaluation of spheroid formation and viability of hepatocytes |
| 11 | Silk woven fibers reinforced silk scaffolds | Amniotic fluid-derived stem cells | The scaffolds were biocompatible and supported cell adhesion and proliferation of stem cells. | Woven silk fabric-reinforced silk nanofibrous scaffolds for regenerating load-bearing soft tissues |
| 12 | Multilayer woven nanofiber yarns of PLA and silk fibroin | Mouse bone marrow-derived mesenchymal stem cells | The scaffolds promoted adhesion and proliferation of stem cells. The stem cells on scaffolds showed efficient osteogenic differentiation along with biomineralization and expression of collagen type I, alkaline phosphatase, and osteocalcin. The scaffolds assisted in bone regeneration upon transplantation in vivo rabbit model. | A biomimetic multilayer nanofiber fabric fabricated by electrospinning and textile technology from polylactic acid and Tussah silk fibroin as a scaffold for bone tissue engineering |
| 13 | Woven melt spun PL and PLCL | Murine bone marrow stromal cells | The scaffolds supported adhesion and viability of the stromal cells. | Design and optimization of a novel bio-loom to weave melt-spun absorbable polymers for bone tissue engineering |
| 14 | Woven PAN nano-micro fibrous yarn (with or without gelatin methacrylate gels) | Human aortic valve interstitial cells | The scaffolds supported adhesion, viability, proliferation of cells along with the expression of heart valve associate markers - vimentin and alpha-smooth muscle actin. | Living nano-micro fibrous woven fabric/hydrogel composite scaffolds for heart valve engineering |
| 15 | PAN-based uniaxially aligned nanofiber yarns (with or without gelatin methacrylate gels) | Human adipose derived stem cells, human aortic valve interstitial cells, and human aortic root smooth muscle cells | The constructs supported adhesion and long-term viability of stem cells. The cell-scaffold constructs exhibited higher efficiency of differentiation towards smooth muscle cells, bone cells. The scaffolds could also be utilized for engineering the heart valves. | Fabrication of Aligned Nanofiber Polymer Yarn Networks for Anisotropic Soft Tissue Scaffolds |
| 16 | Woven PCL/Chitosan/Cellulose nanocrystals composite scaffolds | Human tendon-derived cells and human adipose stem cells | The scaffolds supported the cell adhesion, viability, aligned and elongated morphology along with the expression of tendon-related markers, including Collagen types I and III, tenascin-C, and scleraxis. | 3D Mimicry of Native-Tissue-Fiber Architecture Guides Tendon-Derived Cells and Adipose Stem Cells into Artificial Tendon Constructs |
| 17 | Woven PCL nanofiber yarns with PLA multifilaments | Human adipose-derived stem cells, human tenocytes, and human umbilical vein endothelial cells | The constructs supported adhesion and viability of tenocytes along with the expression of tenocyte specific markers like collagen types I and III, tenascin-C, tenomodulin, and scleraxis. The study also showed the culture of all the cell types, where tri-cultured system promoted early and mature tenogenic differentiation. | Living nanofiber yarn-based woven biotextiles for tendon tissue engineering using cell tri-culture and mechanical stimulation |
| 18 | Woven PLA/hydroxyapatite composite scaffolds. | Human mesenchymal stem cells from bone marrow | The constructs were biocompatible, supported adhesion, viability and efficient osteogenic differentiation of the stem cells. | Osteogenic Differentiation of Human Mesenchymal Stem cells in a 3D Woven Scaffold |
| 19 | Biomineralized woven PLA and silk fibroin nanofiber fabric. | Human mesenchymal stem cells | The scaffolds were non-cytotoxic, supported adhesion, spreading, and viability of the stem cells. The cells on scaffolds differentiated efficiently towards bone lineage. | Biomineralized poly (l-lactic-co-glycolic acid)-tussah silk fibroin nanofiber fabric with hierarchical architecture as a scaffold for bone tissue engineering |
| 20 | Woven PET textiles coated with gelatin | Human induced pluripotent stem cell–derived cardiomyocytes | This scaffold supported the growth of cardiac cells. Cells showed expression of cardiac-specific markers (cardiac alpha-actinin, myosin-binding protein C, and troponin T). | Polyethylene Terephthalate Textiles Enhance the Structural Maturation of Human Induced Pluripotent Stem Cell-Derived Cardiomyocytes |

**Footnotes:** PGA: polyglycolic acid; PET: poly (ethylene therephtalat); PLGA: Poly Lactic-co-Glycolic Acid; PVLA: polyvinylbenzyl-O-beta-D-galactopyranosyl-D-gluconamide; PCL: poly (ε-caprolactone); PL: poly-L-lactide; PLCL: poly-L-lactide-co-ε-caprolactone; PAN: polyacrylonitrile; PLA: polylactic acid.

**References**

[1] A. Neville, R.R. Mather, J.I.B. Wilson, 12 - Characterisation of plasma-treated textiles, in: R.B.T.-P.T. for T. Shishoo (Ed.), Woodhead Publ. Ser. Text., Woodhead Publishing, 2007: pp. 301–315. https://doi.org/https://doi.org/10.1533/9781845692575.2.301.

[2] J. Zhou, D. Cai, Q. Xu, Y. Zhang, F. Fu, H. Diao, X. Liu, Excellent binding effect of l-methionine for immobilizing silver nanoparticles onto cotton fabrics to improve the antibacterial durability against washing, RSC Adv. 8 (2018) 24458–24463. https://doi.org/10.1039/C8RA04401E.

[3] S. Li, T. Xing, Z. Li, G. Chen, Structure and properties of silk grafted with acrylate fluoride monomers by ATRP, Appl. Surf. Sci. 268 (2013) 92–97. https://doi.org/https://doi.org/10.1016/j.apsusc.2012.11.173.

[4] Q. Wang, R. Yanzhang, Y. Wu, H. Zhu, J. Zhang, M. Du, M. Zhang, L. Wang, X. Zhang, X. Liang, Silk-derived graphene-like carbon with high electrocatalytic activity for oxygen reduction reaction, RSC Adv. 6 (2016) 34219–34224. https://doi.org/10.1039/C6RA07075B.

[5] M.K. Khan, J. Luo, Z. Wang, R. Khan, X. Chen, Y. Wan, Alginate dialdehyde meets nylon membrane: a versatile platform for facile and green fabrication of membrane adsorbers, J. Mater. Chem. B. 6 (2018) 1640–1649. https://doi.org/10.1039/C7TB02966G.
